# Network Pharmacology Analysis to Identify Phytochemicals in Traditional Chinese Medicines That May Regulate ACE2 for the Treatment of COVID-19

**DOI:** 10.1155/2020/7493281

**Published:** 2020-11-05

**Authors:** Wenhao Niu, Feng Wu, Haiming Cui, Wenyue Cao, YuChieh Chao, Zonggui Wu, Min Fan, Chun Liang

**Affiliations:** ^1^Department of Cardiology, Shanghai Changzheng Hospital, Second Military Medical University, Shanghai 200001, China; ^2^Department of Cardiology, Yueyang Hospital of Integrated Traditional Chinese and Western Medicine, Affiliated Hospital of Shanghai University of Traditional Chinese Medicine, Shanghai 200080, China; ^3^Department of Ultrasound, Shanghai Chest Hospital, School of Medicine, Shanghai Jiaotong University, Shanghai 200030, China; ^4^Department of Anesthesiology, Shanghai Renji Hospital, School of Medicine, Shanghai Jiaotong University, Shanghai 200120, China

## Abstract

“Three formulas and three medicines,” which include Jinhua Qinggan granule, Lianhua Qingwen capsule/granule, Xuebijing injection, Qingfei Paidu decoction, HuaShiBaiDu formula, and XuanFeiBaiDu granule, have been proven to be effective in curbing coronavirus disease 2019 (COVID-19), according to the State Administration of Traditional Chinese Medicine. The aims of this study were to identify the active components of “Three formulas and three medicines” that can be used to treat COVID-19, determine their mechanism of action via angiotensin-converting enzyme 2 (ACE2) by integrating network pharmacological approaches, and confirm the most effective components for COVID-19 treatment or prevention. We investigated all the compounds present in the aforementioned herbal ingredients. Compounds that could downregulate the transcription factors (TFs) of ACE2 and upregulate miRNAs of ACE2 were screened via a network pharmacology approach. Hepatocyte nuclear factor 4 alpha (HNF4A), peroxisome proliferator-activated receptor gamma (PPARG), hsa-miR-2113, and hsa-miR-421 were found to regulate ACE2. Several compounds, such as quercetin, decreased ACE2 expression by regulating the aforementioned TFs or miRNAs. After comparison with the compounds present in *Glycyrrhiza* Radix et Rhizoma, quercetin, glabridin, and gallic acid present in the herbal formulas and medicines were found to alter ACE2 expression. Gene ontology (GO) and Kyoto Encyclopedia of Genes and Genomes (KEGG) enrichment analysis were used to search for possible molecular mechanisms of these compounds. In conclusion, traditional Chinese medicine (TCM) plays a pivotal role in the prevention and treatment of COVID-19. Quercetin, glabridin, and gallic acid, the active components of recommended TCM formulas and medicines, can inhibit COVID-19 by downregulating ACE2.

## 1. Introduction

The severe acute respiratory syndrome coronavirus 2 (SARS-CoV-2) that causes coronavirus disease 2019 (COVID-19) has rapidly spread worldwide [1]. According to data from the World Health Organization, since the midnight of May 14, 2020, approximately 4,346,487 cases of COVID-19 have been confirmed in 213 countries, with 293,494 deaths. At present, there is no cure for COVID-19. Further studies are warranted to assess the therapeutic effects of vaccines or specific medicines. Traditional Chinese medicine (TCM) is being preferred for treating viral diseases. TCM was used widely and presented promising results when SARS (severe acute respiratory syndrome) and H1N1 influenza were prevalent [2, 3]. TCM substantially improved the symptoms, such as cough, fever, and phlegm [4–6]. Professor Fan Min, a corresponding author of this article, went to Wuhan to take part in the fight against COVID-19 as the director of the C7 ward of Leishenshan Hospital. At the hospital, all patients in the C7 ward were treated with integrated Chinese and western medicine, and 70% of these patients were treated with TCM alone. All the patients were discharged in good health. Among 91.5% of patients treated with TCM and western medicine, the cure rates were above 90%, according to the National Health Commission report [7, 8]. Therefore, TCM treatment can be used to treat COVID-19. Currently, seven versions of the diagnosis and treatment guidelines for COVID-19 have been published by the National Health Commission of China, and various formulas and three medicines have been proven to be effective [4, 9–11]. The State Administration of Traditional Chinese Medicine has proven that the “Three formulas and three medicines,” which include Jinhua Qinggan granule, Lianhua Qingwen capsule/granule, Xuebijing injection, lung cleansing and detoxifying decoction, HuaShiBaiDu formula, and XuanFeiBaiDu granule, are effective against COVID-19.

Nevertheless, the application of TCM is limited worldwide [12], because the underlying mechanisms of action of these drugs are complex and undefined (e.g., lack of data from randomized controlled trials and unclear molecular mechanisms) [13]. Hence, TCM approved by the Chinese Government has been mainly used for the prevention of COVID-19. However, a recent systematic review indicated that TCM can be used as an alternative approach for preventing COVID-19 in vulnerable populations, based on previous reports on TCM use for SARS and H1NI influenza prevention [14, 15].

The angiotensin-converting enzyme 2 (ACE2) can bind to the receptor-binding domain (RBD) of spike glycoprotein, which is responsible for the entry of coronaviruses into the host cells [5, 16]. Therefore, ACE2 can be regarded as an important intervention target for COVID-19 [17]. Therefore, this study focused on active components of TCM on ACE2 and aimed to determine the efficacy of “Three formulas and three medicines” in the treatment of COVID-19 via network pharmacology. We identified the most efficient components and used them to treat or prevent COVID-19.

## 2. Materials and Methods

### 2.1. Identification of Ingredients of TCM

The following TCMs were analyzed: Jinhua Qinggan granules (Juxiechang Pharmaceutical Co. Ltd., Beijing); National medicine approval Z20160001): Jinyin hua (金银花, *Lonicerae Japonicae* Flos, honeysuckle); Shigao (石膏, Gypsum fibrosum moles, Gypsum fibrosum); Ma huang (麻黄, *Ephedra herba*, Ephedra); Kuxing ren (苦杏仁, Amygdalus Communis Vas, Armeniacae Semen Amarum); Huangqin (黄芩, *Scutellariae* Radix, *Scutellaria baicalensis*); Lianqiao (连翘, Forsythiae Fructus, *Fructus forsythiae*); Zhebeimu (浙贝母, *Fritillariae thunbergii* Bulbus, Thunberg Fritillary Bulb); Zhimu (知母, Anemarrhenae Rhizoma, Rhizoma Anemarrhenae); Niubangzi (牛蒡子, Fructus Arctii, Arctii Fructus); Qinghao (青蒿, *Artemisia annua* L., *Artemisiae annua* herba); Bohe (薄荷, *Menthae* Herba, *Menthae haplocalycis* Herba); Gancao (甘草, Licorice, Liquorice); suggested use: one dose per day, boiled with water, twice per day. The treatment course includes six doses. Lianhua Qingwen capsules (Shijiazhuang Yiling Pharmaceutical Co., Ltd; National medicine approval Z20040063): Lianqiao (连翘, Forsythiae Fructus, Fructus Forsythiae); Jinyinhua (金银花, *Lonicerae japonicae* Flos, Honeysuckle); Zhimahuang (炙麻黄, Ephedra, Ephedra); Kuxingren (苦杏仁, *Amygdalus communis* Vas, Armeniacae Semen Amarum); Shigao (石膏, *Gypsum* moles fibrosum, *Gypsum fibrosum*); Banlangen (板蓝根, Isatidis Radix, Radix Isatidis); Mianmaguanzhong (绵马贯众, Male fern rhizome, Male fern rhizome); Yuxingcao (鱼腥草, Houttuynia Herba, *Houttuynia cordata* Thunb); Guanghuoxiang (广藿香, *Pogostemon cablin* (Blanco) Benth, Patchouli); Dahuang (大黄, Rhei Radix Et Rhizoma, Chinese rhubarb); Hongjingtian (红景天, Rhodiola, Rhodiola); Bohenao (薄荷脑, Menthol, Menthol); Gancao (甘草, Licorice, Liquorice); suggested use: four pills at a time, thrice per day, oral administration for 6 days. Xuebijing injection (Tianjin Hongri Pharmaceutical Co., Ltd.; National medicine approval Z20040033): Honghua (红花, *Carthami flos*, Safflower); Chishao (赤芍, Radix Paeoniae Rubra, Red paeony root); Chunaqiong (川穹, Chuanxiong rhizome, Sichuan lovase rhizome); Danshen (丹参, Salviae Miltiorrhizae Radix et Rhizoma, Dan-Shen Root), Danggui (当归, *Angelicae sinensis* Radix, *Angelica sinensis*); suggested use: intravenous infusion of 50 mL Xuebijing injection plus 100 mL saline to be administered within 30–40 min, twice per day. Qingfei Paidu decoction: 9 g Ma huang (麻黄, Ephedra Herba, Ephedra); 6 g Zhigancao (炙甘草, *Glycyrrhizae*, Radix *Glycyrrhizae Preparata*); 9 g Kuxingren (苦杏仁, *Amygdalus communis* Vas, Armeniacae Semen Amarum); 15–30 g Shengshigao (生石膏, Gypsum, Gypsum); 9 g Guizhi (桂枝, Cinnamomi Ramulus, Ramulus cinnamomi); 9 g Zexie (泽泻, Alismatis Rhizoma, *Alisma orientale*); 9 g Zhuling (猪苓, Polyporus, Polyporus); 9 g Baizhu (白术, *Atractylodis macrocephalae* Rhizoma, Largehead Atractylodes Rh); 15 g Fuling (茯苓, Poria, Indian Buead Tuckahoe); 16 g Chaihu (柴胡, Bupleuri Radix; Chinese Thorowax Root); 6 g Huangqin (黄芩, *Scutellariae* Radix, *Scutellaria baicalensis*); 9 g Jiangbanxia (姜半夏; Pinelliae Rhizoma Praeparatum cum Zingibere et Alumine; Rhizome *Pinelliae preparata*); 9 g Shengjiang (生姜, Zingiber officinale Roscoe, Ginger); 9 g Ziyuan (紫苑, Asteris Radix et Rhizoma, Tatarian Aster Root); 9 g Kuandonghua (款冬花, Farfarae Flos; Flos Farfaraes); 9 g Shegan (射干, Belamcandae Rhizome, Rhizoma Belamcandae); 6 g Xixin (细辛, Asari Radix Et Rhizoma, Manchurian wildginger); 12 g Shanyao (山药, Rhizoma Dioscoreae, Rhizoma Dioscoreae); 6 g Zhishi (枳实, Aurantii Fructus Immaturus, Fructus Aurantii Immaturus); 6 g Chenpi (陈皮, *Citrus reticulata*, tangerine peel); 9 g Huoxiang (藿香, Herba Agastachis, Ageratum); suggested use: 1 dose per day, boiled with water, twice per day in the morning and evening. Three doses are a course of treatment. XuanFeiBaiDu granule: 6 g Shengmahuang (生麻黄, Ephedrae Herba, Chinese Ephedra Herb); 15 g Kuxingren (苦杏仁, *Amygdalus communis* Vas, Armeniacae Semen Amarum); 30 g Shengshigao (生石膏, Gypsum, Gypsum); 30 g Shengyiyiren (生薏苡仁, Semen Coicis, Coix Seed); 10 g Maocangshu (茅苍术, Rhizoma *Areactylodis lanceae*, swordlike Atractylodes rhizome); 15 g Guanghuoxiang (广藿香, *Pogostemon cablin* (Blanco) Benth, Patchouli); 12 g Qinghaocao (青蒿草, *Artemisia Annua* L., *Artemisiae Annua* Herba); 20 g Huzhang (虎杖, Polygoni Cuspidati Rhizoma Et Radix, *Polygonum cuspidatum*); 30 g Mabiancao (马鞭草, Verbenae Herb, Herba Verbenae); 30 g Ganmaogen (干茅根, Radix Couchgrass, Couchgrass root); 15 g Tinglizi (葶苈子, Lepidii Semen Descurainiae Semen, pepperweed seed); 15 g Huajuhong (化橘红, *Citri grandis* Exocarpium, Pummelo Peel); 10 g Shenggancao (生甘草, Glycyrrhizae, raw licorice); suggested use: 1 dose per day, boiled with 400 mL water, twice per day in the morning and evening. HuaShiBaiDu formula: 6 g Shengmahuang (生麻黄, Ephedrae Herba, Chinese Ephedra herb); 9 g Xingren (杏仁, *Amygdalus communis* Vas, Apricot kernel); 15 g Shengshigao (生石膏, Gypsum, Gypsum); 3 g Gancao (甘草, Licorice, Liquorice); 10 g Huoxiang (藿香, Herba Agastachis, Ageratum); 10 g Houpu (厚朴, *Magnolia officinalis* Rehd Et Wils, Cortex *Magnolia officinalis*); 15 g Cangzhu (仓术, *Atractylodes lancea* (Thunb.) Dc, Rhizoma Atractylodis); 10 g Caoguo (草果, Amomum Tsao-Ko Crevostet, Tsaoko Amomum Fruit); 9 g Fabanxia (法半夏, Rhizoma Pinelliae, Rhizoma Pinelliae Preparatum); 15 g Fuling (茯苓, Poria, Indian Buead Tuckahoe); 5 g Shengdahuang (生大黄, Rhei Radix Et Rhizoma, Chinese rhubarb); 10 g Shenghuangqi (生黄芪, Astragali Radix, Milkvetch Root); 10 g Tinglizi (葶苈子, Lepidii Semen Descurainiae Semen, pepperweed seed); 10 g Chishao (赤芍, Radix Paeoniae Rubra, red paeony root); suggested use: 1–2 doses per day, boiled with 100–200 mL water, two or four times per day, via oral or nasal administration. These medicines were formulated based on traditional medicine guidelines and identified using the Chinese Clinical Trial Registry (ChiCTR), updated on April 30, 2020. The active compounds of these ingredients were identified using the Traditional Chinese Medicine Systems Pharmacology (TCMSP, http://www.tcmspw.com/tcmsp.php) database [18].

### 2.2. Screening Strategy for Transcription Factors and miRNAs of ACE2

Transcription factors (TFs) are a group of proteins that can bind to a gene promoter and modulate its expression. As small, noncoding RNA molecules, microRNAs (miRNAs) can suppress gene expression by binding to the 3'-UTR of their mRNAs. TFs and miRNAs are both important regulators of gene expression.

TFs and miRNAs of ACE2 were identified using NetworkAnalyst (http://www.networkanalyst.ca) and miRWalk3.0 (mirwalk.umm.uni-heidelberg.de). The active compounds pertaining to TFs and miRNAs of ACE2 were obtained from the TCMSP database and via text mining, respectively [19–21]. Subsequently, compounds that could downregulate the TFs of ACE2 or upregulate miRNAs of ACE2 were screened [19, 21–31]. The pharmacokinetic and structural parameters (oral bioavailability (OB), drug-likeness (DL), blood-brain barrier (BBB) permeability, half-life (HL), and the number of rotatable bonds (RBN)) of the compounds were obtained from the TCMSP database and DrugBank (http://www.drugbank.ca) database.

### 2.3. Network Construction and Analysis

Considering the substantial progress in bioinformatics, network pharmacology has become a useful tool to visualize and characterize the biological processes and pathways regulated by TCM based on numerous databases. To further characterize the molecular mechanism and topological structure of medicines/formulas, compounds, TFs/miRNAs, and ACE2, interaction networks were built and visualized using Cytoscape 3.7.2, a common tool for network interaction research (http://cytosacpe.org/) [32]. In these graphical networks, the medicine/formula, compounds, TFs/miRNAs, and ACE2 were expressed as nodes, and their interactions were expressed as edges.

### 2.4. Gene Ontology and Pathway Enrichment Analysis for Active Compounds

Metascape is a search engine for bioinformatic analysis. It integrates several authoritative data resources, such as GO, KEGG, UniProt, and DrugBank, to provide researchers with comprehensive and detailed information about genes. In the present study, Metascape (http://www.metascape.org) and Cytoscape 3.7.2 were used to analyze the biological processes and pathways of genes related to chenodeoxycholic acid, quercetin, genistein, chrysoeriol, wedelolactone, tectorigenin, glabridin, isoarnebin 4, yohimbine, fisetin, Rg1, and gallic acid. These genes were retrieved from the TCMSP database and confirmed using the DrugBank database. All gene names were standardized using UniProtKB (http://www.uniprot.org/) database [33].

## 3. Results

### 3.1. Several Compounds Decreased ACE2 Expression via Regulation of TFs or miRNAs of ACE2

**Figure 1:**
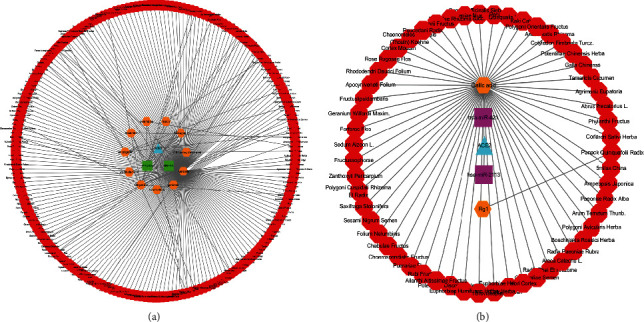
The Herbs-Compounds-TFs/miRNAs-ACE2 networks. (a) The Herbs-Compounds-TFs-ACE2 network was constructed by using Cytoscape 3.7.2. The results revealed that HNF4A could be downregulated by chenodeoxycholic acid, and PPARG could be downregulated by quercetin, genistein, chrysoeriol, wedelolactone, tectorigenin, glabridin, isoarnebin 4, yohimbine, and fisetin. (b) The Herbs-Compounds-miRNAs-ACE2 network was constructed by using Cytoscape 3.7.2. Rg1 and gallic acid could upregulate hsa-miR-2113 and hsa-miR-421, respectively. The red octagons represent herbs, and the orange hexagons represent compounds. The green squares represent TFs, and the pink rectangles represent miRNAs. The blue triangles represent ACE2.

After TFs and miRNAs of ACE2 were downloaded from the database, compounds that could downregulate TFs of ACE2 or upregulate miRNAs of ACE2 were identified. As summarized in Table 1, HNF4A could be downregulated by chenodeoxycholic acid. PPARG could be downregulated by multiple compounds, including quercetin, genistein, chrysoeriol, wedelolactone, tectorigenin, glabridin, isoarnebin 4, yohimbine, and fisetin. Rg1 and gallic acid could upregulate hsa-miR-2113 and hsa-miR-421, respectively. As shown in Figure 1, we used Cytoscape 3.7.2 to visualize the topological structure of the compounds, TFs/miRNAs, and ACE2 interaction networks. The complex relationship among these networks is clearly illustrated.

**Figure 2:**
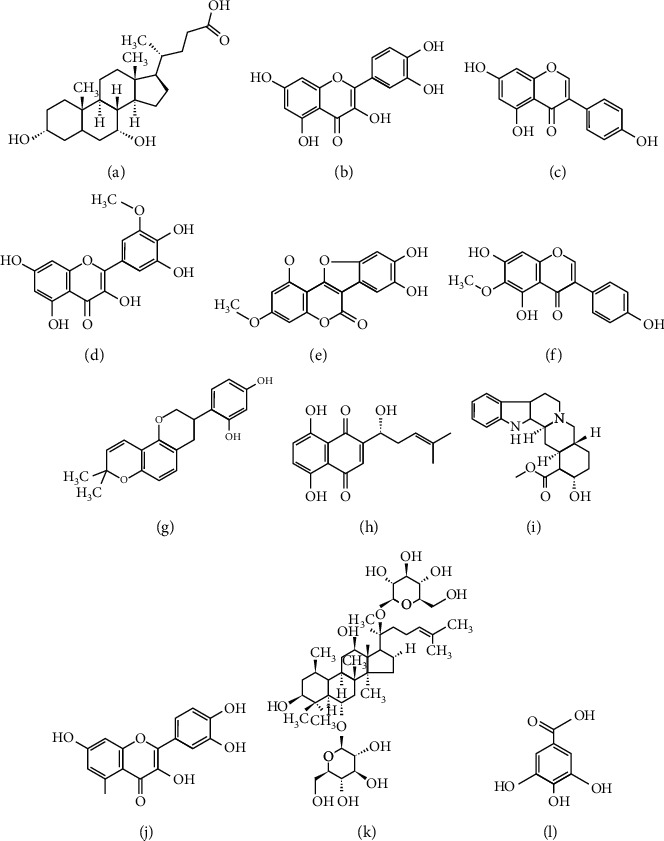
Chemical structures of (a) chenodeoxycholic acid, (c) genistein, (b) quercetin, (d) chrysoeriol, (e) wedelolactone, (f) tectorigenin, (g) glabridin, (h) isoarnebin 4, (i) yohimbine, (j) fisetin, (k) Rg1, and (l) gallic acid.

**Figure 3:**
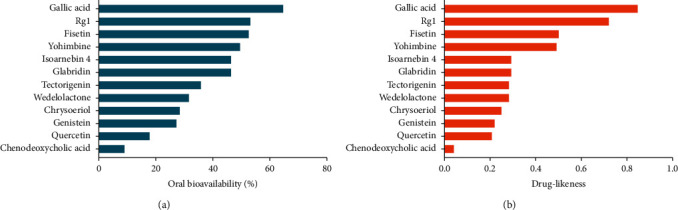
Compound ranking by oral bioavailability and drug-likeness. (a) Compounds ranked according to oral bioavailability. (b) Compounds ranked according to drug-likeness. Compounds were ranked according to OB and DL, which may help in the selection of drugs for COVID-19 treatment.

The structures of the aforementioned compounds are illustrated in Figure 2, and the pharmacokinetic and structural parameters, namely, OB, DL, BBB, HL, and RBN, of the compounds are summarized in Table 2. Of these parameters, OB and DL are considered the most important. OB (F%) refers to the fraction of an orally administered drug that reaches systemic circulation. DL is defined as a complex balance of various molecular properties and structural features that determine whether a particular molecule is similar to a known drug. As shown in Figure 3, we ranked compounds according to OB and DL to determine compounds that may be helpful to develop drugs for COVID-19 treatment. Collectively, these results indicate that a selected group of active substances could target the TFs or miRNAs of ACE2, thereby subsequently reducing the expression of ACE2.

Jinhua Qinggan granules, Lianhua Qingwen capsules, Xuebijing injection, Qingfei Paidu decoction, XuanFeiBaiDu granule, and HuaShiBaiDu formula may exert therapeutic effects via regulation of the TFs or miRNAs of ACE2.

We investigated whether the aforementioned medicines and formulas could decrease ACE2 expression by regulating the TFs or miRNAs of ACE2. After comparing the compounds of the herbs present in the medicines and formulas with those listed in Table 1, we identified common compounds (Table 3). Quercetin, glabridin, and gallic acid were the most frequently used compounds in the medicines and formulas examined.

**Figure 4:**
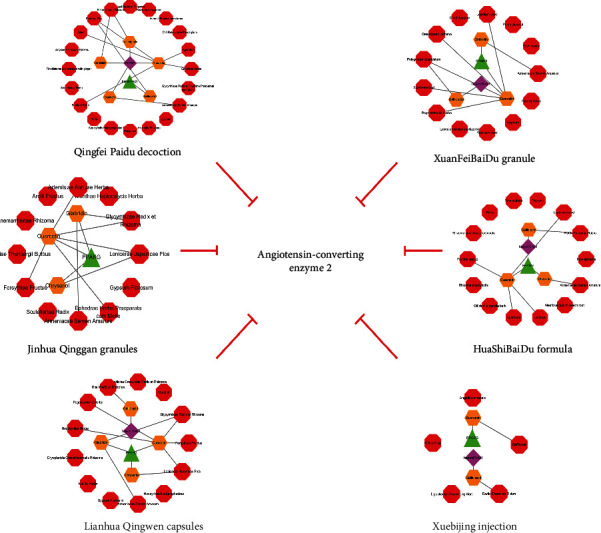
The Herbs-Compounds-TFs/miRNAs-ACE2 networks of Jinhua Qinggan granules, Lianhua Qingwen capsules, Xuebijing injection, Qingfei Paidu decoction, XuanFeiBaiDu granules, and HuaShiBaiDu formula. The results indicated that these medicines/formulas could exert therapeutic effects via regulation of TFs or miRNAs of ACE2. The red octagons represent herbs, the orange hexagons represent compounds, the green triangles represent TF, and the pink diamonds represent miRNA.

Of the 12 compounds, 6 were present in the medicines/formulas. As summarized in Table 3, three compounds were present in Qinggan granules (quercetin, glabridin, and chrysoeriol), three in Lianhua Qingwen capsules (quercetin, glabridin, and chrysoeriol), two in Xuebijing injection (quercetin and gallic acid), five in Qingfei Paidu decoction (quercetin, genistein, tectorigenin, glabridin, and gallic acid), three in XuanFeiBaiDu granule (quercetin, glabridin, and gallic acid), and three in HuaShiBaiDu formula (quercetin, glabridin, and gallic acid**)**. These results suggest that these medicines and formulas could exert therapeutic effects via regulation of TFs or miRNAs of ACE2 (Figure 4).

### 3.2. Metascape and Cytoscape Visualizations of the Biological Processes and Pathways of Compounds

**Figure 5:**
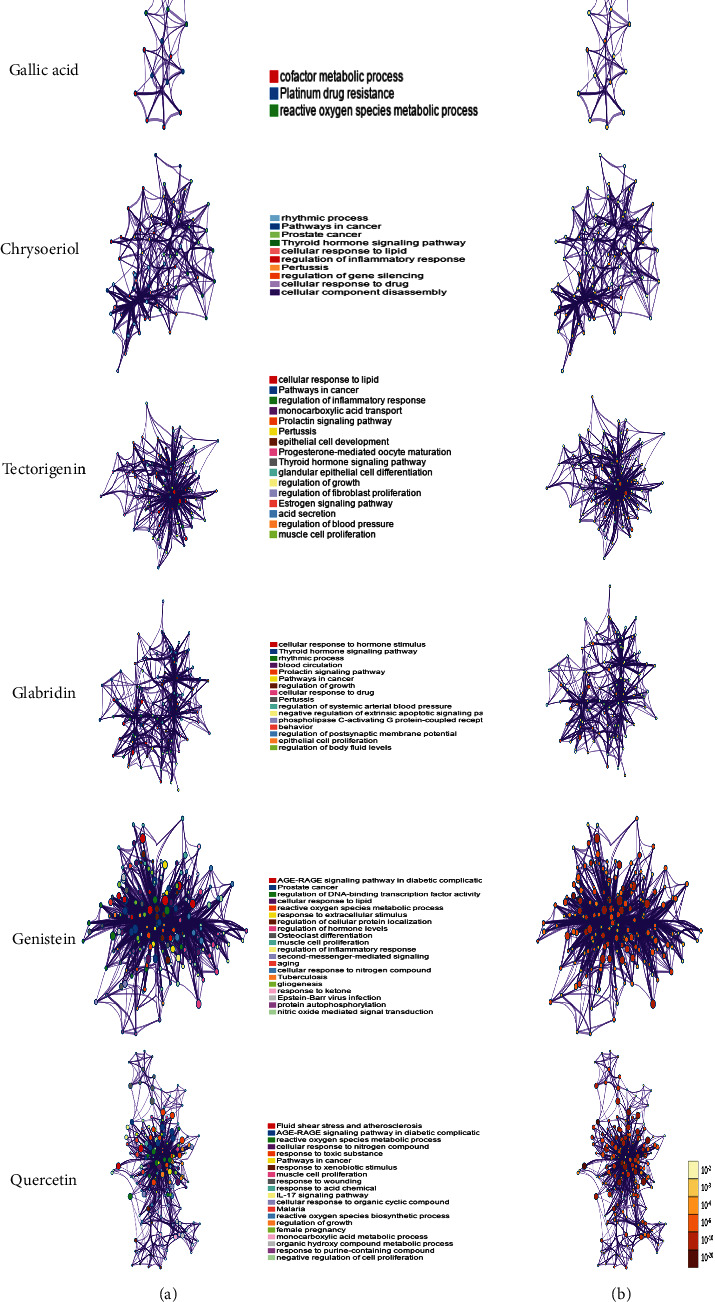
Biological processes and pathways impacted by quercetin, genistein, chrysoeriol, tectorigenin, glabridin, and gallic acid, analyzed by Metascape. (a) Network of enriched terms colored by cluster identity. Nodes that share the same cluster identity are close to each other. (b) Network of enriched terms colored by *p* value; more genes tended to result in a more significant *p* value.

**Figure 6:**
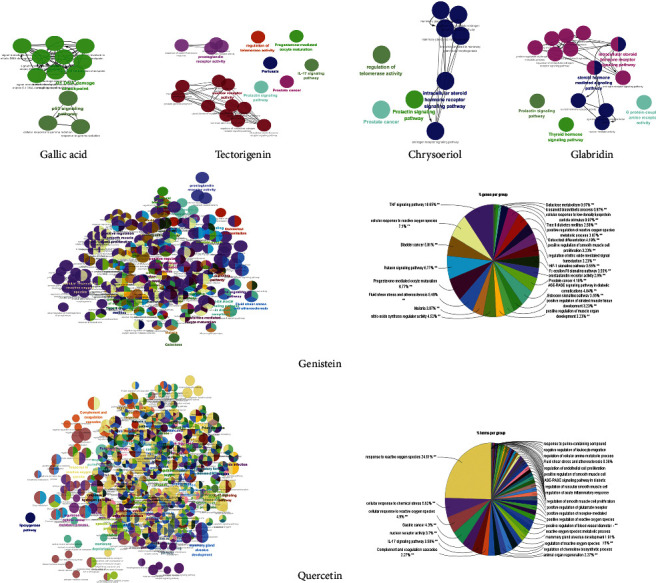
Biological processes and pathways impacted by quercetin, genistein, chrysoeriol, tectorigenin, glabridin, and gallic acid, analyzed by Cytoscape 3.7.2. ClueGO plug-in was used to analyze the interaction networks of enriched biological processes and pathways. The multicolored dots indicate that multiple biological processes and pathways are involved.

We downloaded the genes associated with the compounds from the TCMSP database (Table 4). Metascape and Cytoscape 3.7.2 were then used to analyze the biological processes and pathways of the compounds. As illustrated in Figure 5, in the Metascape analysis, fluid shear stress and atherosclerosis, AGE-RAGE signaling pathway in diabetic complications, cellular response to nitrogen compounds, and the pathway in cancer were the mainly involved biological processes and pathways following quercetin administration. Cellular response to hormone stimulus, thyroid hormone signaling pathway, and rhythmic process were mainly associated with glabridin, and cofactor metabolic processes, platinum drug resistance, and reactive oxygen species (ROS) metabolic processes were mainly associated with gallic acid. As visualized by Cytoscape (Figure 6), quercetin was mainly associated with genes involved in response to ROS, cellular response to chemical stress, and cellular response to ROS. These results suggest that quercetin, glabridin, and gallic acid could regulate multiple biological processes and pathways that could alleviate COVID-19 symptoms.

**Figure 7:**
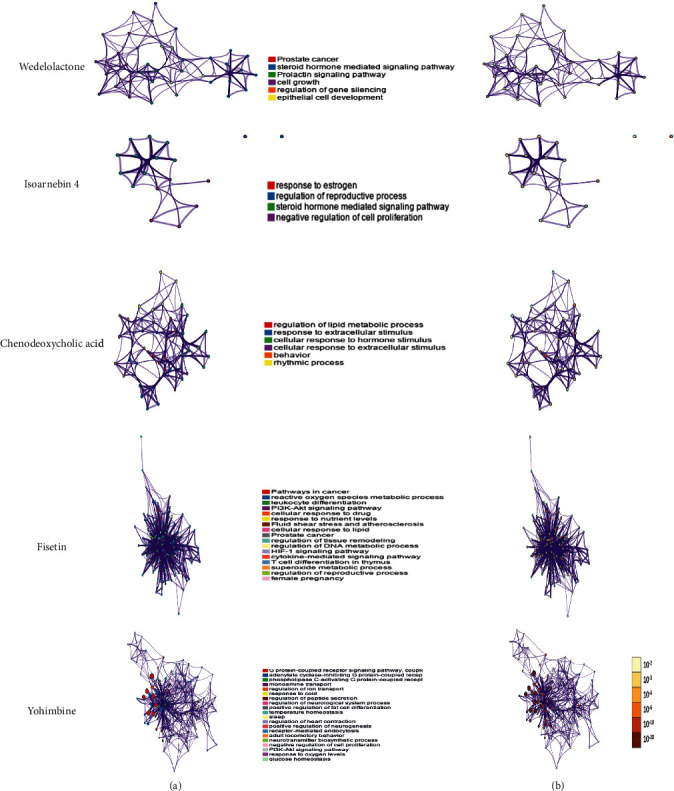
Biological processes and pathways impacted by wedelolactone, isoarnebin 4, chenodeoxycholic acid, fisetin, and yohimbine, as analyzed by Metascape. (a) The network of enriched terms colored by cluster identity. Nodes that share the same cluster identity are close to each other. (b) The network of enriched terms colored by *p* value; more genes tended to result in a more significant *p* value.

**Figure 8:**
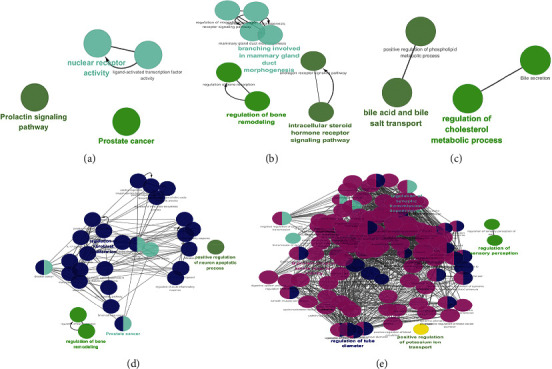
Biological processes and pathways impacted by (a) wedelolactone, (b) isoarnebin 4, (c) chenodeoxycholic acid, (d) fisetin, and (e) yohimbine, as analyzed by Cytoscape 3.7.2. The ClueGO plug-in was used to analyze the interaction networks of the enriched biological processes and pathways. The multicolored dots indicate that multiple biological processes and pathways are involved.

Moreover, the biological processes and pathways affected by chenodeoxycholic acid, wedelolactone, isoarnebin 4, yohimbine, and fisetin were analyzed; these results are presented in Figures 7 and 8. Although these compounds were not identified in the medicines or formulas, they may be used as potential treatment options for COVID-19.

## 4. Discussion

In the past 20 years, epidemics such as SARS and Middle East respiratory syndrome (MERS), caused by coronavirus, have severely impacted the life expectancy and the quality-of-life of people worldwide. In December 2019 in Wuhan, Hubei Province, China, the Chinese Center for Disease Control and Prevention (CDC) reported an unexplained case of pneumonia, which was finally diagnosed as a novel coronavirus disease (COVID-19).

Although several medicines have revealed protective effects in vulnerable populations [34, 35], the remarkable curative effect of TCM on COVID-19 cannot be ignored. Previous studies have reported that Qingfei Paidu decoction, Shufeng Jiedu capsules, Lianhua Qingwen capsules, Maxing Shigan decoction, Huoxiang Zhengqi capsules, and Jinhua Qinggan granules contributed to the recovery from COVID-19 [10, 14]; however, the mechanisms of action of these TCM formulations remain poorly understood.

ACE2 is crucial in the negative regulation of the renin-angiotensin system (RAS) [36] and was recently identified as the main functional receptor of SARS-CoV-2 [37, 38]. Considering the wide distribution of ACE2, patients with COVID-19 are at risk of multiple organ dysfunction [39–41]. Therefore, ACE2 is an important intervention target for COVID-19.

Numerous studies have been conducted on phytochemicals and ACE2 for the treatment of COVID-19. A previous study has identified several small-drug molecules of natural origin, such as quercetin and curcumin, which could impede the coronavirus S-protein:ACE2 interface-ligand binding complex through a molecular docking study, which subsequently decreased the possibility of viral entry into the body [5]. In other studies, by means of molecular docking, constituents such as flavonoids may serve as potential inhibitors to target ACE2 and disturb the interaction between the virus and ACE2 [42]. Furthermore, the main protease (Mpro) of COVID-19 could also be targeted by several phytochemicals, which exert antiviral effects and protect the respiratory system [43].

It has been reported that several TCMs can reduce the expression of ACE2 and, thus, exert antiviral effects [44]; however, therapies that only target ACE2 directly are insufficient. Targeting the upstream regulators of ACE2, such as TFs and miRNAs, is crucial; however, the effects of TCM on the regulation of TFs and miRNAs of ACE2 have not been investigated.

In the present study, we initially identified 12 compounds that downregulated TFs or upregulated miRNAs of ACE2 and then selected quercetin, glabridin, gallic acid, genistein, chrysoeriol, and tectorigenin via screening. As a naturally occurring flavonoid, quercetin is abundant in the roots, stems, leaves, flowers, and fruits of plants. Quercetin exerts various pharmacological effects in several diseases. Substantial evidence highlights that quercetin can alleviate the progress of diabetic encephalopathy and protect human oral keratinocytes through its antioxidant, anti-inflammatory, and antiapoptotic activities [45, 46]. Furthermore, it can also prevent platelet aggregation, lower blood pressure, and regulate blood lipids, which protect against I/R-induced myocardial injury [47, 48]. Recently, a study using supercomputer-based drug-docking analysis of the COVID-19 viral spike protein confirmed that quercetin could be an important binding partner that hampers the interactions between the virus and ACE2 [49].

Glabridin is a bioactive ingredient in licorice. Studies have reported that glabridin can exert antioxidant activity via inhibition of ROS production and upregulation of superoxide dismutase (SOD) [50]. Moreover, it can exert an anti-inflammatory effect via inhibition of inflammatory signaling pathways and factors [51]. Furthermore, cancer stem cell-like properties can be suppressed by glabridin [52]. Gallic acid is a natural polyphenolic compound that is abundant in Galla Chinensis, Radix Sanguisorbae, and other herbs. Gallic acid is mostly absorbed by the human body and is transformed into 4-O-methylgallic acid in the body [53]. Previous studies have reported that gallic acid has antiallergic, anti-inflammatory, and antiviral effects [54].

Similar to quercetin and glabridin, chrysoeriol is a naturally occurring flavonoid. Previous studies reported that it may decrease blood endotoxins and TNF-*α* levels in rats [55]. Genistein has a strong antioxidant effect and may serve as an inhibitor of protein tyrosine kinase. It plays a pivotal role in radiation protection by scavenging ROS and other free radicals [56]. Tectorigenin is one of the main components of Belamcanda rhizome and Puerariae Flos. Several studies have investigated the effects of tectorigenin on antitumor and anti-inflammatory activities [57–59]; however, no research is presently available on glabridin, gallic acid, chrysoeriol, genistein, or tectorigenin, and ACE2. Considering their oral bioavailability and drug-likeness, these may be efficiently used in COVID-19 treatment.

It is very difficult to convey the experiences of treating COVID-19 with TCM to people worldwide. Although the efficacy of TCM in treating COVID-19 is known, the associated mechanism remains unclear; however, the safety of TCM is questionable. In particular, ephedra is banned in the US owing to toxicity and it is not considered safe by the European Food Safety Authority (EFSA). Chinese medicine believes that ephedra can eliminate phlegm and relieve cough, nourish Yin and choking lung, and transport water and clear the damp. In clinical practice, Chinese medicines containing ephedra are often used for treating cold, cough, asthma, expectoration, and other symptoms. Moreover, based on the theory of TCM, the rational combination of drugs will neutralize their toxicity and exert a strong therapeutic effect; thus, the toxicity of ephedra in drugs will be substantially reduced. Zhong Nanshan, an academic researcher of the Chinese Academy of Engineering, confirms that the oral administration of a combination of conventional treatment and Lianhua Qingwen capsule for 14 days can remarkably improve fever, fatigue, cough, and other clinical symptoms associated with COVID-19, markedly improve the pulmonary imaging lesions, reduce the duration of symptoms, improve the clinical cure rate, and repress the deterioration of COVID-19, with a good safety record; however, multicenter randomized controlled trials with a large sample size are required to confirm the efficacy and safety of TCM in the treatment of COVID-19.

## 5. Conclusions

Our study indicated that quercetin, glabridin, and gallic acid, three potential compounds for the treatment of COVID-19, may decrease the expression of ACE2 via regulation of TFs or miRNAs of ACE2 and subsequently exert an antiviral effect.

## Figures and Tables

**Table 1:** Regulatory relationships between the compounds and TFs/miRNAs of ACE2.

Compound	Chemical formula	Target	Description
Chenodeoxycholic acid	C_24_H_40_O_4_	HNF4A	**↓**
Genistein	C_15_H_10_O_5_	PPARG	**↓**
Quercetin	C_15_H_10_O_7_	PPARG	**↓**
Chrysoeriol	C_16_H_12_O_6_	PPARG	**↓**
Wedelolactone	C_16_H_10_O_7_	PPARG	**↓**
Tectorigenin	C_16_H_12_O_6_	PPARG	**↓**
Glabridin	C_20_H_20_O_4_	PPARG	**↓**
Isoarnebin 4	C_16_H_16_O_5_	PPARG	**↓**
Yohimbine	C_21_H_26_N_2_O_3_	PPARG	**↓**
Fisetin	C_15_H_10_O_6_	PPARG	**↓**
Rg1	C_42_H_72_O_14_	hsa-miR-2113	**↑**
Gallic acid	C_7_H_6_O_5_	hsa-miR-421	**↑**

**↓**: downregulation; **↑**: upregulation

**Table 2:** Pharmacokinetic and structural parameters of compounds.

Compound	OB	DL	BBB	HL	RBN
Chenodeoxycholic acid	9.03	0.28	−0.94	—	4
Quercetin	17.93	0.21	−0.77	14.4	1
Genistein	27.17	0.69	−0.4	—	1
Chrysoeriol	28.41	0.27	−0.53	16.31	2
Wedelolactone	31.69	0.04	−0.45	9.61	1
Tectorigenin	35.85	0.27	−0.37	—	2
Glabridin	46.42	0.81	0.36	0.03	1
Isoarnebin 4	46.43	0.28	−0.65	29.33	3
Yohimbine	49.6	0.48	−0.02	9.37	2
Fisetin	52.6	0.24	−0.69	15.06	1
Rg1	53.25	0.47	−3.41	—	10
Gallic acid	64.79	0.2	−0.54	11.78	1

OB : oral bioavailability; DL : drug-likeness; BBB : blood-brain barrier; HL : half-life; RBN : number of rotatable bonds.

**Table 3:** The distribution of the compounds of interest in medicines and formulas.

Compound	Jinhua Qinggan granules	Lianhua Qingwen capsules	Xuebijing injection	Qingfei Paidu decoction	XuanFeiBaiDu granules	HuaShiBaiDu formula
Chenodeoxycholic acid						
Quercetin	√	√	√	√	√	√
Genistein				√		
Chrysoeriol	√	√				
Wedelolactone						
Tectorigenin				√		
Glabridin	√	√		√	√	√
Isoarnebin 4						
Yohimbine						
Fisetin						
Rg1						
Gallic acid			√	√	√	√

Quercetin, genistein, chrysoeriol, tectorigenin, glabridin, and gallic acid were found in medicines and formulas analyzed, wherein quercetin, glabridin, and gallic acid were the most frequently used compounds.

**Table 4:** Genes altered by quercetin, genistein, chrysoeriol, tectorigenin, glabridin, and gallic acid.

Compounds	Genes
Quercetin	*SERPIND1; MAPK1; HMOX1; MMP2; PLAT; PON1; PON2; MPO; CCL2; COL1A1; SULT1E1; IL2; IFNG; ODC1; CTSD; GSTM2; AHR; KCNH2; IL1B; EGF; RB1; TP53; SOD1; EGFR; GJA1; AKR1B1; PPARG; VCAM1; MMP3; ACHE; INSR; COL3A1; ADRB2; PRSS1; GSTM1; NQO1; MMP1; ACPP; HSPA5; SELE; PTGDR2; F3; HSP90AA1; CYP3A4; CYP1A2; PTGER3; DPP4; TOP1; CD40LG; JUN; PLAU; ALOX5; AR; F7; THBD; MAOB; XDH; CDK1; BCL2; GSTP1; IL6; GABRA1; TOP2B; NCOA2; SCN5A; FAM213B; RXRA; POR; COX14; MGAM; VEGFA; ACACA*

Genistein	*MAPK1; GCK; CCL2; SULT1E1; MAPK14; MAPK3; IL1B; CDK2; TP53; EGFR; SERPING1; AKR1B1; CDK1; INS; BCL2; ESR1; FAM213B; LDLR; VEGFA; BTK; PPARG; HMGCR; VCAM1; HPGD; GLB1; PRSS1; NOS2; SELE; PTGDR2; RAB11B; HDAC6; HSP90AA1; KCNJ11; CD40LG; PTGFR; JUN; AR; SYK*

Chrysoeriol	*MAPK14; CDK2; SERPING1; PPARG; PRSS1; GSK3B; NOS2; PCP4; PTGDR2; HSP90AA1; DPP4; AR; NCOA1; NCOA2; ESR1; FAM213B*

Tectorigenin	*MAPK14; IL1B; CDK2; CCNA2; SERPING1; PPARG; PRSS1; GSK3B; NOS2; PCP4; PTGDR2; HSP90AA1; ESR2; PTGER3; SPDEF; AR; PAK7; NCOA1; ESR1; FAM213B; RXRA*

Glabridin	*MAPK14; CDK2; CCNA2; SERPING1; PPARG; ACHE; ADRB2; CHRM1; ADRA1B; PRSS1; GSK3B; NOS2; PCP4; PTGDR2; ESR2; RXRB; AR; PAK7; NCOA1; NCOA2; ESR1; SCN5A*

Gallic acid	*TOP2B; PGR; PTGDR2; FASN; MGST1; MAOB; TP53; PTPN21; HSP90AA1*

These genes were obtained from the TCMSP database and confirmed in the DrugBank database. All gene names were standardized by using the UniProtKB database for *Homo sapiens*.

## Data Availability

The data used to support the findings of this study are included within the article. Additional data can be made available from the corresponding author upon request.

## Additional Points

*Limitations*. The study only analyzed the active components of “three formulas and three medicines,” which were used to treat COVID-19 and their mechanisms of action via ACE2; however, SARS-CoV-2 could cause illness via several pathways; TCM presents multitarget function. We consider quercetin, glabridin, and gallic acid as three potential drugs for COVID-19 that act by inhibiting ACE2; however, animal models or human studies to confirm the same are warranted.
